# Surgical Repair of Ectopia Cordis in a Sub-Saharan African Country (Benin)

**DOI:** 10.1055/a-2698-3658

**Published:** 2025-09-18

**Authors:** Abdel Kémal Bori Bata, Désiré Nékoua, Ahmad Ibrahim, Boris Gogan, Eugène Zoumenou

**Affiliations:** 1 University Visceral Surgery Clinic, Faculty of Health Sciences, University of Abomey-Calavi, Cotonou, Benin; 2 University Cardiology Clinic, Faculty of Health Sciences, University of Abomey-Calavi, Cotonou, Benin; 3 Multipurpose University Clinic of Anesthesia and Resuscitation, Faculty of Health Sciences, University of Abomey-Calavi, Cotonou, Benin; 4 University Clinic of Pediatric Surgery, Faculty of Health Sciences, University of Abomey-Calavi, Cotonou, Benin

**Keywords:** ectopia cordis, sternal agenesis, surgical correction, sub-Saharan Africa

## Abstract

**Background:**

Ectopia cordis (EC) is an exceptionally rare congenital anomaly with a poor prognosis, particularly in resource-limited settings.

**Case Description:**

We present a newborn with complete thoracic EC who underwent surgical correction on day 5 of life. Although cardiac repositioning and soft tissue coverage were achieved, the infant died 72 hours postoperatively from septic shock, reflecting the significant challenges faced in such contexts.

**Conclusion:**

This case underscores the urgent need for improved antenatal care and technical resources to enhance outcomes for complex congenital anomalies in sub-Saharan Africa.

## Introduction

Ectopia cordis (EC) is an extremely rare congenital defect, occurring in approximately 5 to 8 per million live births. It is defined by partial or complete extrusion of the heart outside the thoracic cavity. 1 2 EC presents in four anatomical variants: Cervical, thoracic, thoracoabdominal, and abdominal, often with associated cardiac or midline anomalies. 2 3 4 Despite advances in neonatal surgery, management remains challenging, and prognosis is typically poor. 2 We report a case of surgically managed EC during the neonatal period in a low-resource African setting.

## Case Presentation

A female neonate, admitted at 2 hours of life, presented with a congenital anterior thoracic wall defect. Antenatal care began at 29 weeks of gestation, with only one obstetric ultrasound, which did not reveal any anomalies. The 39-year-old mother had not received folic acid supplementation. No consanguinity or teratogenic exposure was reported. The infant, sixth in the sibling order, was delivered vaginally at 36 weeks of gestation in a level I peripheral health care facility.

**Fig. 1:**
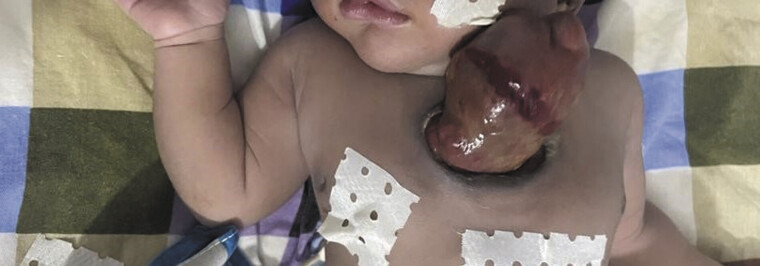
Clinical image showing complete thoracic ectopia cordis.

At examination, she appeared in moderate general condition, with normal anthropometric parameters (weight: 3,095 g, length: 50 cm, head circumference: 34 cm). She exhibited respiratory distress (Respiratory Rate [RR]: 66 cpm, Peripheral capillary oxygen saturation [SpO _2_ ]: 91%) without cyanosis. A 6-cm anterior thoracic wall defect exposed a pulsatile, tachycardic (170 bpm), uncovered heart with a superiorly oriented apex. A circumferential wound was noted on the posterior myocardial surface ( Fig. 1 ). Neurological exam was unremarkable, and no other anomalies were clinically evident.

**Fig. 2:**
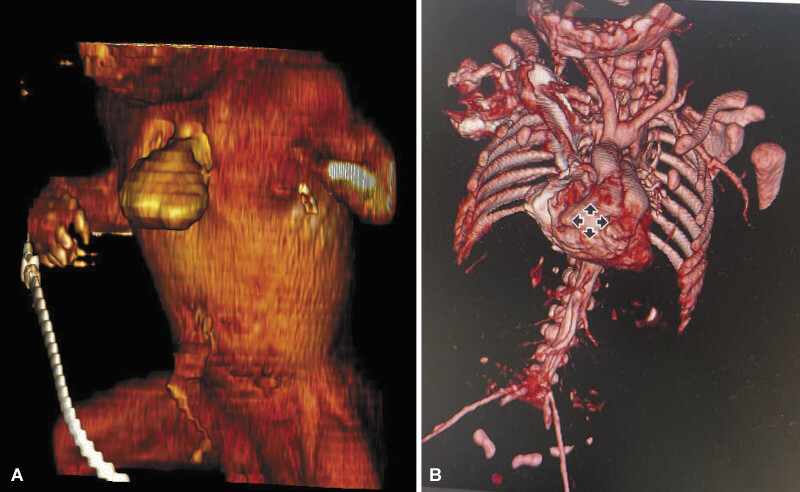
Three-dimensional CT reconstructions of complete thoracic ectopia cordis. ( **A** ) Anterior volume-rendered view showing complete extrusion of the heart outside the thoracic cavity through a large midline anterior wall defect with sternal agenesis. ( **B** ) Oblique view centered on the vascular axis, depicting the ectopic heart, thoracoabdominal vascular structures, and preserved posterior rib arches.

Echocardiography revealed no structural cardiac malformation. Chest CT scan confirmed complete sternal agenesis with thoracic EC ( Fig. 2 ). Inflammatory markers were elevated. The patient underwent stabilization, prevention of hypothermia and hypoglycemia, aseptic management with non-abrasive protective dressing of the exposed heart, and antibiotic therapy based on ceftriaxone.

**Fig. 3:**
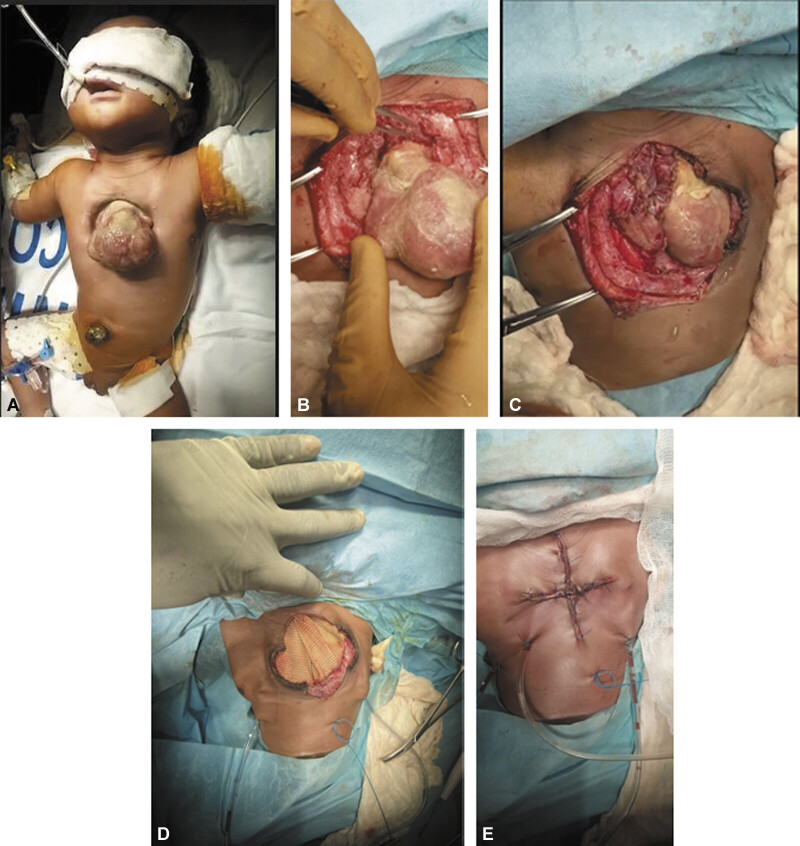
Stepwise surgical repair of complete thoracic ectopia cordis. ( **A** ) Preoperative view showing the completely exposed heart protruding through a large anterior thoracic wall defect. ( **B** ) Initial dissection and mobilization of the great vessels, preparing for cardiac reintegration. ( **C** ) Successful reduction of the heart into the thoracic cavity without hemodynamic compromise. ( **D** ) A tailored Dacron patch was sutured to the native pericardium to create a protective pericardial pouch. ( **E** ) Final cross-shaped skin closure over suction drains, completing the single-stage repair.

On day 5 of life, under general anesthesia, a median incision extending above and below the cardiac exit site was performed. Dissection and mobilization of the superior and inferior vena cava, aorta, and supra-aortic trunks were achieved. The left pleura was opened, and the heart was successfully repositioned into the thoracic cavity with hemodynamic stability. A pericardial pouch was constructed using a Dacron patch. Thoracic wall approximation allowed for a cross-shaped skin closure ( Fig. 3 ). Following cardiac reintegration and skin closure, intraoperative monitoring parameters, including filling pressures, remained within normal limits. No intraoperative complications were observed. The neonate was subsequently transferred to the neonatal intensive care unit under assisted ventilation. A ceramic prosthetic reconstruction was planned at age 3. After an initial 48-hour period of clinical stability, the patient experienced marked clinical deterioration, characterized by fever, hypotension, oliguria, and hypoglycemia. Blood cultures grew a strain of *Acinetobacter* spp. resistant to ceftriaxone but sensitive to piperacillin–tazobactam and carbapenems, prompting targeted adjustment of antibiotic therapy. Despite intensive resuscitation measures, including the use of inotropes, the neonate died 72 hours postoperatively.

## Discussion

The etiology of EC remains unclear, though several hypotheses exist: Failure of cardiac descent and midline fusion, chorionic rupture with constrictive bands, or *BMP2* gene deficiency affecting ventral wall development. 2 EC is categorized by heart location: Cervical (5%), thoracic (65%), abdominal (10%), and thoracoabdominal (20%). 3 Our patient had complete thoracic EC.

There is a female predominance in reported cases, consistent with our observation. 5 In sub-Saharan Africa, diagnosis is generally made at birth, unlike in high-income countries, where prenatal detection may occur as early as 10 weeks of gestation, facilitating early multidisciplinary planning or pregnancy termination. 2

EC is frequently associated with additional malformations, such as intracardiac defects or part of the Cantrell pentalogy. 2 Our case was isolated. Literature reports that in 41% of cases, the heart is uncovered, 31% are covered by a membrane, and 27% by skin. 3 In our case, the heart was entirely exposed.

Surgical goals include soft tissue coverage, intracardiac anomaly correction, cardiac repositioning, and thoracic reconstruction, which may be staged. 6 Staged repair is generally preferred to mitigate the risk of cardiac compression or pulmonary hypoplasia. Thoracic and intracardiac repair often follows repositioning. 6 7 Early neonatal intervention is advised, especially in cases with exposed myocardium. It is generally accepted that early complete cardiac reintegration is desirable, as it provides adequate anatomical protection and restores optimal physiological function, provided that the thoracic cavity offers sufficient space. This consideration guided our decision to pursue this strategy in our patient. The second stage, involving thoracic wall reconstruction, relies on the use of musculocutaneous flaps or synthetic and autologous materials. Autologous grafts are favored due to reduced infection risk and adaptability to growth. 6 7 In low-resource settings, reconstructive options remain limited.

In sub-Saharan Africa, most reported cases are stillbirths or deaths before intervention. 3 4 Postoperative mortality is commonly due to hemodynamic instability or compression-related complications. Complete thoracic EC is associated with a high postoperative mortality rate, primarily because reintegration of the heart into the thoracic cavity frequently causes distortion of arterial or venous connections. In addition, tight skin closure further increases ventricular filling pressures, thereby compromising diastolic function. 2 Furthermore, postoperative sepsis may occur despite adequate surgical coverage, particularly in cases of complete cardiac externalization associated with delayed surgical intervention, as was the case in our patient. 8 In this case, hemodynamic failure due to compression or vascular distortion appears unlikely. The thoracic capacity observed intraoperatively allowed for cardiac reintegration without significant constraint, and hemodynamic status remained stable during the first 48 postoperative hours. Septic shock appears to be the most plausible etiology, given the presence of preoperative sepsis, confirmed by a positive blood culture for *Acinetobacter* on day 6 of life, following a febrile episode that occurred 24 hours before death.

Importantly, the 5-day delay between birth and surgical intervention may have contributed to infectious progression. Delayed surgery is a recognized prognostic factor, with the literature reporting an average delay of 5 hours in survivors versus 25 hours in non-survivors. 8 This complication further supports the critical need for prenatal diagnosis, enabling optimal delivery planning—preferably by cesarean section—earlier surgical management, improved hemodynamic stability, reduced infection risk, and ultimately, better survival outcomes.

Despite surgical advances, survival remains uncertain. In summary, the prognosis is generally poor even after surgery and depends on EC type, thoracic cavity capacity, heart coverage, time to surgery, and associated anomalies. 3 8 Nevertheless, some successful postoperative cases have been documented, with reported postoperative survival rates in developed countries ranging from 50 to 70%. 2

This case underscores the potential viability of staged repair, even in low-resource environments, provided that neonatal support is adequate. It highlights the urgent need for improved antenatal diagnostic tools and neonatal intensive care resources in developing countries.

## Conclusion

EC is a rare congenital anomaly with a generally poor prognosis, especially in its complete thoracic form. In sub-Saharan Africa, diagnosis is often delayed until birth due to limited prenatal imaging. This case highlights the urgent need to strengthen antenatal services through early prenatal detection, timely referral to specialized centers, and well-planned surgical intervention.
